# Molecular Mobility in Oriented and Unoriented Membranes Based on Poly[2-(Aziridin-1-yl)ethanol]

**DOI:** 10.3390/polym13071060

**Published:** 2021-03-28

**Authors:** Roberto Teruel-Juanes, Krzysztof Artur Bogdanowicz, Jose D. Badia, Victor Sáenz de Juano-Arbona, Robert Graf, Jose A. Reina, Marta Giamberini, Amparo Ribes-Greus

**Affiliations:** 1 Institute of Technology of Materials (ITM), Universitat Politècnica de València (UPV), Camí de Vera s/n, 46022 València, Spain; r.teruel@upvnet.upv.es (R.T.-J.); vsaenzdejuano@gmail.com (V.S.d.J.-A.); 2 Department of Chemical Engineering, Universitat Rovira i Virgili (URV), Av. Països Catalans, 26, 43007 Tarragona, Spain; bogdanowicz@witi.wroc.pl (K.A.B.); marta.giamberini@urv.cat (M.G.); 3 Polymer Technology and Sustainability Group, Department of Chemical Engineering, School of Engineering, Universitat de València (UVEG), Avinguda de la Universitat s/n, 46100 Burjassot, Spain; jose.badia@uv.es; 4 Max Planck Institute for Polymer Research, Ackermannweg 10, 55021 Mainz, Germany; graf@mpip-mainz.mpg.de; 5 Department of Analytical Chemistry and Organic Chemistry, Universitat Rovira i Virgili (URV), C/Marcel·lí Domingo s/n, 43007 Tarragona, Spain; joseantonio.reina@urv.cat

**Keywords:** dielectric relaxation spectra, macromolecular cooperativity, segmental dynamics, dendronic liquid crystal membranes, poly[2-(aziridin-1-yl)ethanol] (PAZE)

## Abstract

Unoriented and oriented membranes based on dendronized polymers and copolymers obtained by chemical modification of poly[2-(aziridin-1-yl) ethanol] (PAZE) with the dendron 3,4,5-tris[4-(*n*-dodecan-1-yloxy)benzyloxy]benzoate were considered. DSC, XRD, CP-MAS NMR and DETA, contribute to characterize the tendency to crystallize, the molecular mobility of the benzyloxy substituent, the dendritic liquid crystalline group and the clearing transition. The orientation of the mesogenic chain somewhat hindered this molecular motion, especially in the full substituted PAZE. The fragility, free volume and thermal expansion coefficients of these membranes near the glass transition are related to the orientation and the addition of the dendritic groups. PAZE-based membranes combine both order and mobility on a supramolecular and macroscopic level, controlled by the dendritic group and the thermal orientation, and open the possibility of preparing membranes with proper channel mobility that promotes selective ionic transport.

## 1. Introduction

Liquid crystals (LCs) can be considered as prototypical self-organizing molecular materials because their structural organization stands between the isotropic liquid and the strongly organized crystalline state. Factors that control the arrangement of various LC phases primarily are molecular shape, micro-segregation of incompatible parts, specific molecular interaction, self-assembly, and self-organization. Hierarchical self-assembly in LCs offers a strategy for obtaining nanostructured mesophases and, consequently, it has potential interest for components in membranes where the supramolecular structure of the polymer plays an important role in the selective transport processes of ions, liquid, gases or charges [1,2,3,4,5].

The dendronized polymers and copolymers poly[2-(aziridin-1-yl) ethanol] (PAZE) with the dendron 3,4,5-tris[4-(n-dodecan-1-yloxy)benzyloxy] benzoate exhibit a liquid crystalline columnar structure [6,7,8,9,10,11,12,13], due to a self-assembly process driven by the exo-recognition of the dendritic moieties [14]. As a result, their main chains form an arrangement of columnar channels and the presence of the basic nitrogen in the inner part facilitates the interaction with protons and other cations. The presence of the oriented channels is expected to enhance the selective transport processes of these membranes, without the need for water attendance. As a matter of fact, the preparation of oriented membranes based on these polymers or copolymers was reported, which showed promising transport properties and selectivity. Moreover, low water uptake was reported [6,7,8,10,11]. For these reasons, these materials could be successfully applied in Direct Methanol Fuel Cells (DMFCs) or artificial photosynthesis devices. On the other hand, the applicability of these membranes depends on their ability of rapid and selective transfer of cations, as well as on their mechanical properties; both aspects are mainly determined by the polymer mobility and orientation. Therefore, it might be desirable to get further insight into the dynamics of large supramolecular building blocks of these materials, in order to improve their self-assembly and optimize their performances [14,15,16].

Differential Scanning Calorimetry (DSC), X-ray diffraction (XRD) and ^13^C Cross Polarization Magic Angle Spinning (CP-MAS) NMR contribute to characterize these materials as far as their structure and tendency to crystallize is concerned. Two factors must be taken into account, in order to predict the final properties of these dendronized materials: the chain flexibility, which leads to an easier arrangement into crystalline (but not necessarily stable) domains in the native polymers, and the region regularity which, as usual, is decisive for the formation of a stable crystalline phase.

On the other hand, in order to get the proper channel orientation for ion transport, the membranes based on these materials need to be thermally treated: this previous thermal orientation during the preparation of the membrane could slightly modify the columnar self-assembly, and consequently, the molecular mobility. In this sense, dielectric spectroscopy is also a very useful technique to assess the structure and in particular the dynamics of these large supramolecular systems, by considering the response to an electrical perturbation field over a wide frequency range at different temperatures [17,18,19,20].

Thus, the aim of the current work was to study the effect of the partial and full modification with lateral dendritic groups and their orientation on molecular dynamics of polymers and copolymers of poly[2-(aziridin-1-yl) ethanol] (PAZE) dendronized with 3,4,5-tris[4-(n-dodecan-1-yloxy)benzyloxy] benzoate. A deep characterization of the dielectric relaxation spectrum could contribute to clarify the mobility and the dynamic fragility of these polymers and copolymers. The balance between chain flexibility and structural regularity is key to define the dynamics of these supramolecular systems, which could control the selective transport processes.

## 2. Materials and Methods

### 2.1. Materials

**Figure 1:**
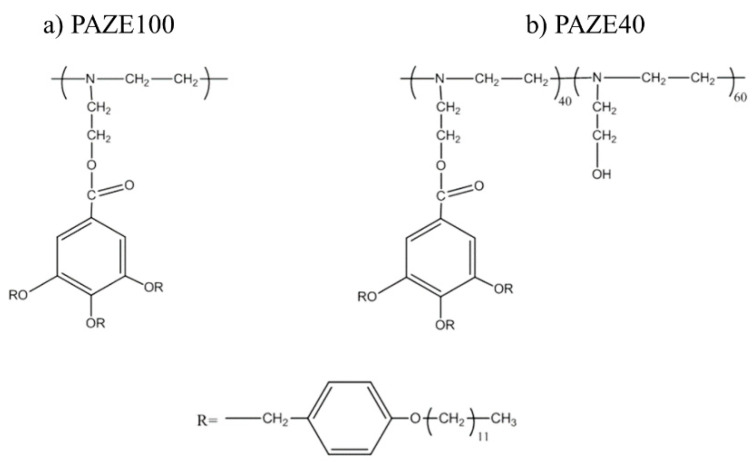
Structures of poly[2-(aziridin-1-yl) ethanol] (PAZE) polymer and copolymer.

The starting material which was subsequently dendronized was poly[2-(aziridin-1-yl)ethanol] (PAZE), of our synthesis (polymerization degree 44 repeating units, as determined by SEC-MALLS) [21]. The polymers were obtained by grafting PAZE with the dendron 3,4,5-tris[4-(n-dodecan-1-yloxy)benzyloxy]benzoic acid as previously described^6^. In this paper, we investigated the behaviour of the partially dendronized PAZE40 (degree of modification of PAZE equal to 40%) and the fully modified PAZE100, which structures are schematized in Figure 1.

Membranes were prepared out of these polymers by means of phase inversion precipitation onto a Teflon^®^ (Dupont, Wilmington, Delaware, USA) substrate. In this process, a homogeneous polymer solution in THF (30% *w*/*w*) was cast on a Teflon sheet support and immersed in a coagulation bath of Milli-Q water. The solvent diffused into the coagulation bath, while water (non-solvent) diffused into the cast film. After de-mixing of the polymer solution, a solid polymer membrane was finally obtained which was dried overnight at around 293 K room temperature. Oriented membranes were obtained by means of a proper thermal treatment, named baking. This treatment consists of heating the obtained membranes, above the polymer clearing temperature, on a Teflon^®^ support with a Linkam (Linkam Scientific Instruments, Tadworth, UK) TP92 hot stage and maintaining them at this temperature for ten minutes. Subsequently, the membranes were slowly cooled (0.5 K·min^−1^) to an annealing temperature lower than, but close to, the polymer clearing point, and kept at this temperature for 72 h. Finally, they were slowly cooled (0.5 K·min^−1^) to 293 K room temperature and detached from the Teflon^®^ support. Oriented polymer and copolymer membranes are labelled with the suffix–O.

### 2.2. Characterization Techniques

Calorimetric studies of dendronized polymers were performed in aluminium standard 40 mL crucibles without pin (ME-26763) with a Mettler DSC822e thermal analyzer (Mettler Toledo, Columbus, OH, USA) at the heating rate of 10 K/min using about 5 mg of sample, nitrogen as a purge gas (100 mL/min) and liquid nitrogen for the cooling system. The equipment was previously calibrated with indium (429.7 K) and zinc (692.6 K) pearls.

X-ray diffraction (XRD) measurements were performed with a Siemens (Siemens AG, München, Germany) D5000 diffractometer (Bragg–Brentano parafocusing geometry and vertical θ-θ goniometer) fitted with a curved graphite diffracted-beam monochromator, incident and diffracted-beam Soller slits, a 0.06° receiving slit and scintillation counter as a detector. Samples were dissolved in a few drops of THF, placed directly onto a low background Si(510) sample holder for reflection analysis and then the solvent was evaporated before analysis. The X-ray diffractometer was operated at 40 kV and 30 mA to generate CuKα radiation. The angular 2θ diffraction range was between 1 and 40°. The data were collected with an angular step of 0.03° at 6 s per step.

The combination of magic angle spinning (MAS) with the ^13^C cross-polarization (CP) NMR spectra (^1^H MAS and ^13^C CP-MAS) of PAZE100 were recorded in a temperature range from T = 250 K to T = 350 K. The measurements were performed at 25 kHz MAS and 850 MHz ^1^H Larmor frequency using a Bruker (Bruker, Billerica, MA, USA) Avance III console and a commercial double-resonance MAS probe supporting zirconia rotors with 2.5 mm outer diameter. The RF power levels on ^1^H and ^13^C were adjusted to an RF nutation frequency of 100 kHz, corresponding to a 90° pulse length of 2.5 µs. The CP-MAS NMR spectra were acquired with a CP contact pulse length of 1 ms and 2048 transients, using SPINAL64 decoupling during acquisition. The adjusted VT gas temperatures were corrected for frictional heating under fast MAS conditions using the known temperature-dependent chemical shift of lead nitrate, so that the given temperature values reflect the actual sample temperature under the chosen MAS conditions.

Dielectric Thermal Analysis (DETA) was performed to measure the dielectric spectra in the frequency range f = 10^−2^–10^7^ Hz at temperatures from 123 to 393 K, by increasing steps of 10 K, by means of a Dielectric Spectrometer of Novocontrol Technologies (Novocontrol Technologies GmbH and Co. KG, Hundsangen, Germany). The spectra were characterised in terms of the real *ε*′ and imaginary *ε*″ parts of the complex dielectric permittivity ε*, for a given set of temperatures and frequencies [17,19,20,22,23,24,25]. The dielectric spectra were deconvoluted by means of the Charlesworth method [26], by summative Havriliak–Negami (HN) functions [27,28]. Other parameters as the relaxation time *τ*, the values of the dielectric intensity or relaxation strength Δ*ε*, and *a_HN_*, *b_HN_* Havriliak–Negami parameters, which determine the symmetric and asymmetric broadening of the relaxation peak, were also calculated.

The thermal activation of the relaxation times *τ* was characterized in terms of intramolecular or intermolecular segmental motions, by means of Arrhenius or Vogel–Fulcher–Tamman–Hesse functions, respectively [29,30].

Arrhenius equation:

(1) $$f_{max}=\;f_{0}\exp\left(\frac{-E_{a}}{R\;T}\right)$$

where *f_max_* is the maximum frequency; *f*_0_ the Pre-exponential term and *E_a_* the Apparent activation energy.

Vogel–Fulcher–Tamman–Hesse equation:

(2) $$\tau \;\left(T\right)=\;\tau_{0}\exp\left(\frac{B}{T-T_{K}}\right),\;T_{K}\;<\;T_{g}$$

where *B* = D·*T_K_*; D is related to the topology of the theoretical potential energy surface of the system, where fragile systems (D ≤ 6) present high density of minimum energy, contrarily to strong systems (D ≥ 15) which present lower density.

The intermolecular relaxations were characterized in terms of dynamic fragility, free-volume and thermal expansion coefficients [31,32], as has been carried out elsewhere [19,33,34,35].

Fragility index:

(3) $$m=\frac{B\;T_{K}}{ln\left(10\right)\;{\left(T_{G}-T_{K}\right)}^{2}}$$

## 3. Results

### 3.1. Calorimetric, X-ray and NMR Analyses

**Figure 2:**
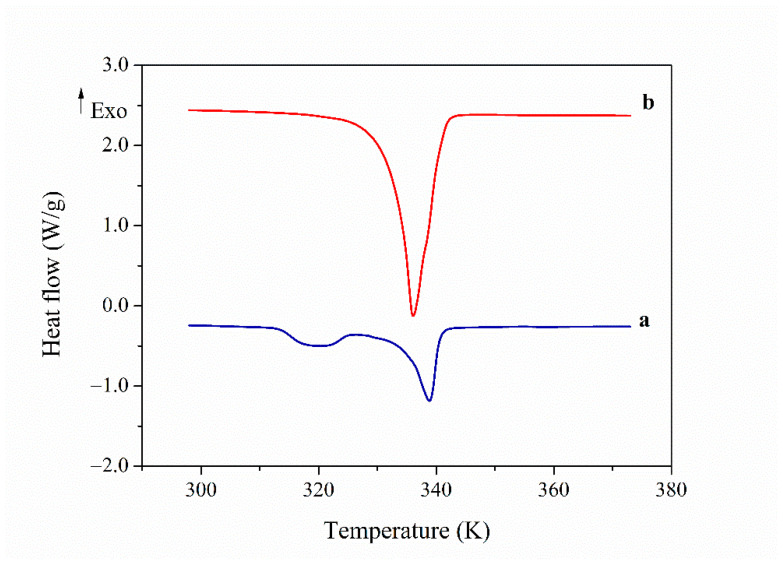
Differential Scanning Calorimetry (DSC) thermogram on first heating of: (**a**) PAZE40; (**b**) PAZE100.

Figure 2 displays the calorimetric thermograms corresponding to the DSC first heating scan of PAZE40 and PAZE100. In the case of the partially modified PAZE40 it is possible to distinguish two endotherms, the first one around 320 K and the other at 340 K. The former may be related to the melting of a small crystalline portion, while the latter relates to the clearing transition, both associated to the lateral mesogenic chains.

However, the thermogram of fully modified PAZE100 shows only a single endotherm at 336 K, which is related to the clearing transition, alike the partially modified membrane. It is noteworthy to highlight that the glass transition could not be visualized in either of the PAZE membranes.

**Figure 3:**
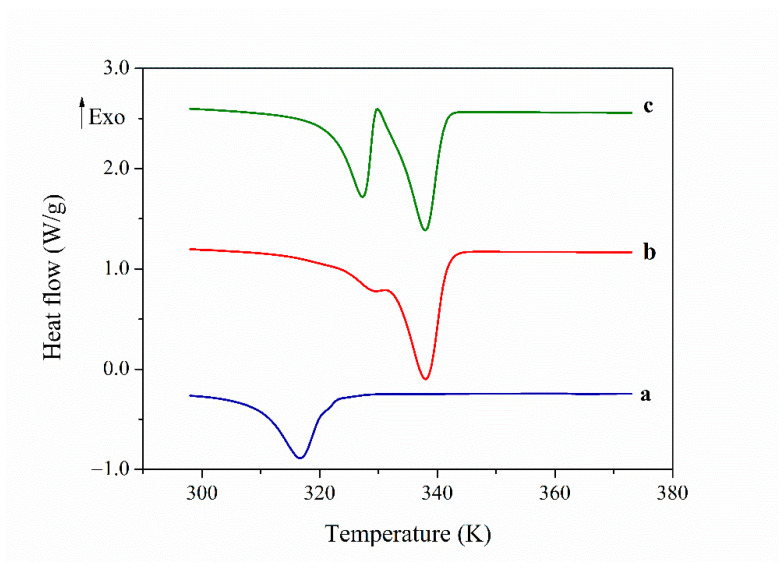
DSC thermogram on second heating of: (**a**) PAZE40; (**b**) PAZE100; (**c**) thermogram of PAZE100 after (**b**) and subsequent annealing 2 h at 318 K.

Figure 3 puts into evidence the different behaviour of both membranes PAZE100 and PAZE40 after a heating process, i.e., in the DSC second heating scan. The thermogram of PAZE100 shows two peaks, one at 340 K, related to the clearing transition with an enthalpy of 33 kJ/g, and the other peak, located around 330 K, partially overlapped, which was increased and better resolved after annealing 2 h at 318 K and suggested the existence of a crystalline portion in the annealed sample.

The thermograms obtained during the second heating scan for PAZE40, show only a single endothermic peak at 316 K, with an enthalpy of 31 kJ/g. The position of this peak, having the results of the first heating scan in mind, may suggest the assignment to the melting of a crystalline portion. However, the observation by Optical Microscopy between Crossed Polars (POM) indicates the breakdown of the mesogenic order and thus attributes this transition to a clearing phenomenon, which has displaced to lower temperature. To verify the origin of these peaks, XRD measurements need to be performed.

**Figure 4:**
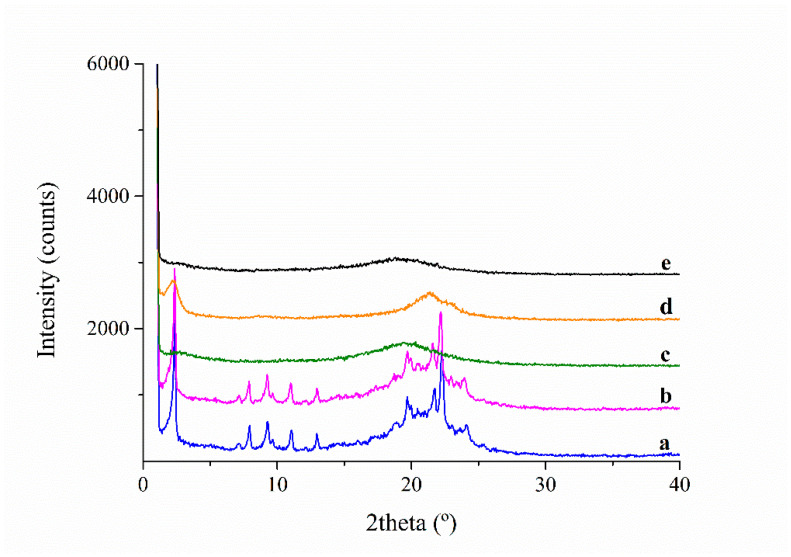
X-ray Diffraction (XRD) pattern of PAZE40: (**a**) native at 294 K; (**b**) after cooling to 223 K; (**c**) after subsequent heating to 353 K; (**d**) after second cooling to 233 K; (**e**) after second heating to 343 K.

**Figure 5:**
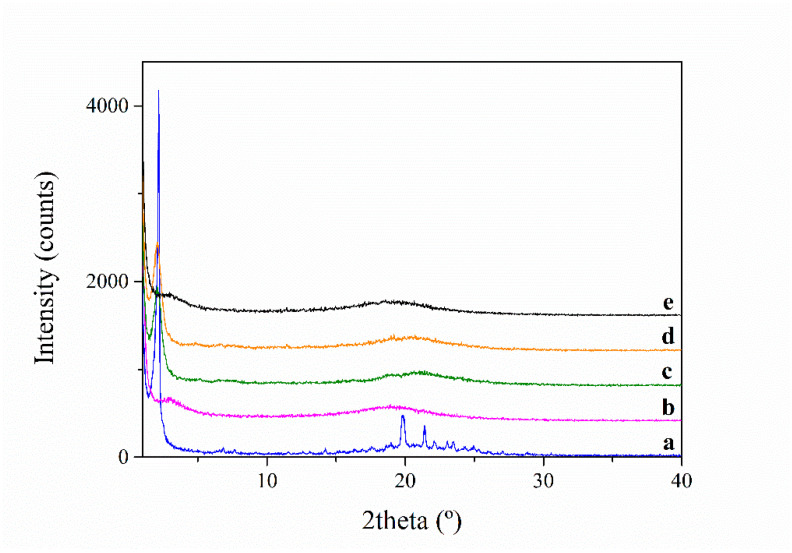
XRD pattern of PAZE100: (**a**) native at 296 K; (**b**) after heating 10 K/min to 353 K; (**c**) after cooling 1 K/min to 272 K; (**d**) after 2 h annealing at 318 K; (**e**) after subsequent heating to 353 K.

X-ray diffraction (XRD) measurements were carried out at room temperature (293K) and the results are plotted in Figure 4 and Figure 5**.** The native PAZE40 exhibits many reflections indicating crystalline contribution, which remained unaltered on cooling to 223 K. On heating to 353 K, all reflection signals disappeared. On cooling back to 233 K, the initial crystallinity was not restored; instead, three very broad reflections at 2θ = 2.0°, 20.7°, 23.4° are observed, corresponding to d = 44 Å, 4.3 Å and 3.8 Å, respectively. They disappeared on heating to 343 K and were attributed to a liquid crystalline columnar phase. These observations suggested that in native PAZE40 a small crystalline portion coexists with a mesophase order. However, when a PAZE40 membrane was heated and subsequently cooled, only the clearing phenomenon associated with the liquid crystalline side chains is restored. These results also confirmed that clearing is the origin of the endothermic peak observed at 316 K in the second heating scan for the PAZE40 membrane.

XRD analyses of PAZE100 performed at different temperatures are shown in Figure 5. The native membrane clearly shows a crystalline structure at 296 K. The analysis performed after heating 1 K/min to 353 K showed just a broad halo around 20°, indicating that the membrane is isotropic at this temperature. Afterwards, it was cooled back 1 K/min to 272 K; nevertheless, the crystalline structure could not be restored by this treatment. On the other hand, a reflection at 2θ = 2° was evident and was attributed to the inter-columnar distance of columnar LC phase 6. However, the asymmetry of the halo located around 20° may suggest a coexistence of the mesophase with crystallinity to a very low extent. Afterwards, XRD was recorded at 318 K, after 2 h annealing at this temperature. In this case, differently from calorimetric analysis, crystallinity could be only guessed as small peaks corresponding to 2θ = 11–12° and 19–20°. After further heating to 353 K, the XRD pattern evidenced that the treatment drove the membrane to the isotropic state again.

As far as the liquid crystalline columnar organization of PAZEs is concerned, in a previous paper, the dimension of the unit cell and the number of repetitive units contained could be calculated [13]. In the case of both PAZE40 and PAZE100, the dimension of the hexagonal unit cell was around 45 Å. Nevertheless, the number of repetitive units in the case of PAZE40 is 10.5, while it was found 4.6 units in the case of PAZE100.

**Figure 6:**
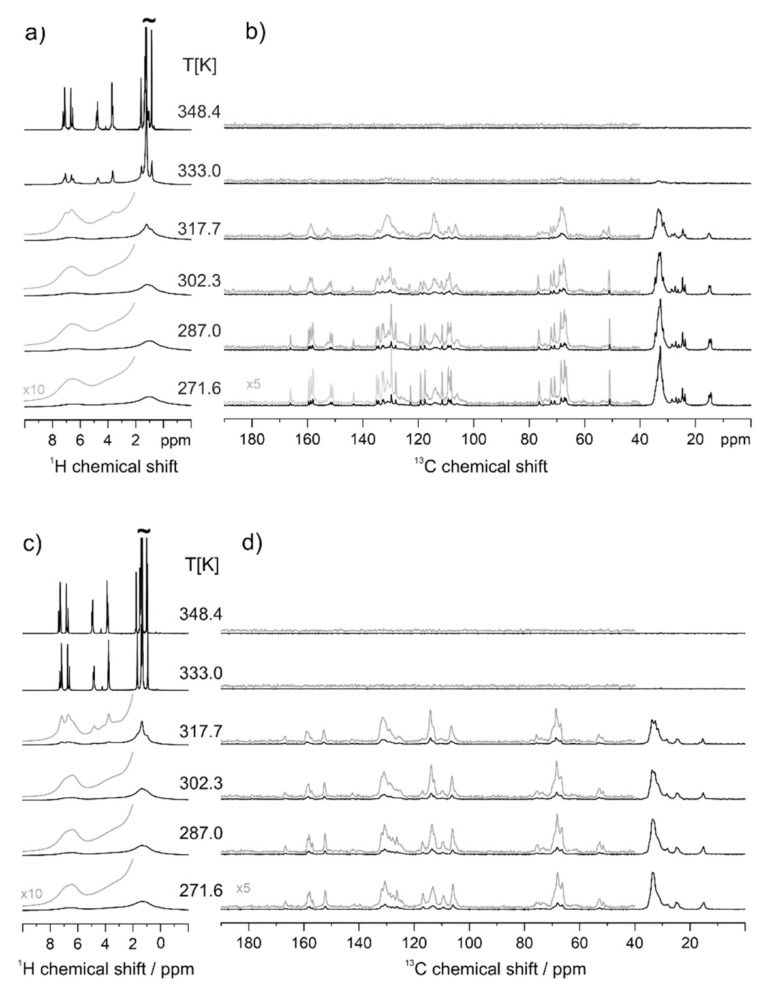
^1^H Magic Angle Spinning (MAS) and ^13^C CP-MAS NMR spectra native PAZE100 (**a**,**b**) and thermal treated PAZE 100 (**c**,**d**) at variable temperatures. The given temperature values are corrected for the frictional heating under fast MAS conditions.

On the other hand, the presence of crystallinity in native PAZE100 could be confirmed by solid-state NMR, which is reported in Figure 6**.** The presence of much higher conformational order in native PAZE100 membrane is evident from the narrow, well defined ^13^C CP-MAS NMR signals in the low-temperature range (Figure 6b), especially in the chemical shift region 100–170 ppm (characteristic of carbonyl and aromatic groups of the side moiety) and 20–34 ppm (alkyl chains of side groups) [13]. At temperatures above T = 290 K (corrected T value = 302 K) these signals broaden and vanish, confirming that melting occurs below this temperature and that crystallinity involves mainly the mesogenic side groups. In addition, the vanishing of all signals in ^13^C CP-MAS spectrum and the simultaneous line narrowing in ^1^H MAS spectra above 333 K (Figure 6a) confirms the occurrence of clearing transition below this temperature.

In the thermally treated PAZE100 sample (Figure 6c,d), the sharp features in the chemical shift range from 100–170 ppm, are only a minor contribution and the broader signals indicate the less defined molecular packing the mesogenic side groups.

**Figure 7:**
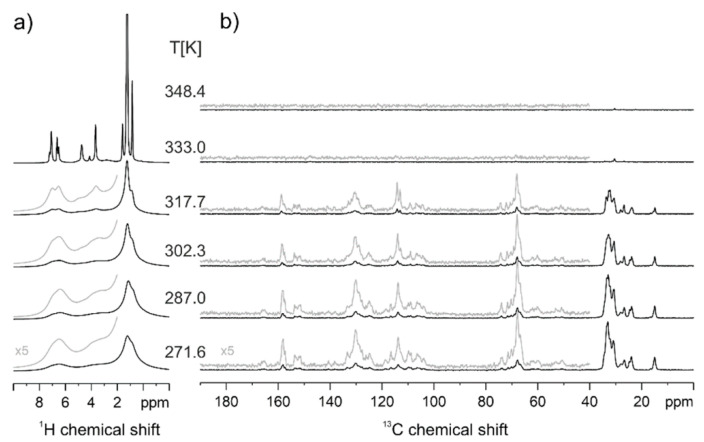
PAZE40 ^1^H MAS (**a**) and ^13^C CP-MAS (**b**) NMR spectra at variable temperature. In brackets, the temperature values corrected for frictional heating under fast MAS conditions are reported.

In solid-state NMR spectra of PAZE40 (Figure 7), only a well-defined transition from a partially ordered mesophase to the isotropic state is observed between T = 318 K and T = 333 K. In the isotropic state, narrow, well-resolved ^1^H MAS signals are observed, and the polarization transfer of the CP-MAS experiment fails, as seen by the vanishing of all ^13^C CP-MAS signals at the same temperature (besides a tiny signal of some main chain methylene units). The transition temperature matches well, the temperature observed in the second heating run of the DSC measurements. In the ^1^H MAS NMR spectra of the partially ordered phase of PAZE40, a gradual signal narrowing with increasing temperature is observed.

One can expect, that the higher the modification degree in PAZE, the stronger the tendency to crystallize. Nevertheless, the presence of the bulky side groups, though leads to a more regular structure, which tends to decrease main chain flexibility. Therefore, in the case of membranes with partial modification degrees such as PAZE40, orientation seems to favor the organization into a more stable LC phase, while crystalline portions tend to disappear. On the contrary, in the case of fully modified PAZE100, high regularity of the structure could favor organization of the polymer into crystalline domains; nevertheless, rigidity of the structure prevents the arrangement of long-range crystalline structures from occurring directly on cooling from the molten state.

### 3.2. Dielectric Response of PAZE Polymer and Copolymers

The above results indicate that the thermal treatment, applied during the preparation of the oriented membranes, could slightly modify the columnar self-assembly and consequently, the molecular mobility. Therefore, deep characterization of the dielectric relaxation spectrum could contribute to clarify the relevance of the two factors that may define the final properties of these materials: the chain flexibility, which leads to an easier arrangement, and the structure regularity, necessary for the formation of a stable crystalline phase.

Dielectric measurements were performed on unoriented and oriented PAZE40 and PAZE100 membranes. The dielectric relaxation spectra of polymer and copolymer based on poly[2-(aziridin-1-yl)ethanol] (PAZE) were measured in terms of the real *ε*′ and imaginary *ε*″ components of the complex dielectric permittivity ε* respectively, in a wide range of frequencies and temperatures. The differences in dielectric behaviour of the unoriented (PAZE100 and PAZE40) and oriented, (PAZE100-O and PAZE40-O) membranes, were analyzed.

**Figure 8:**
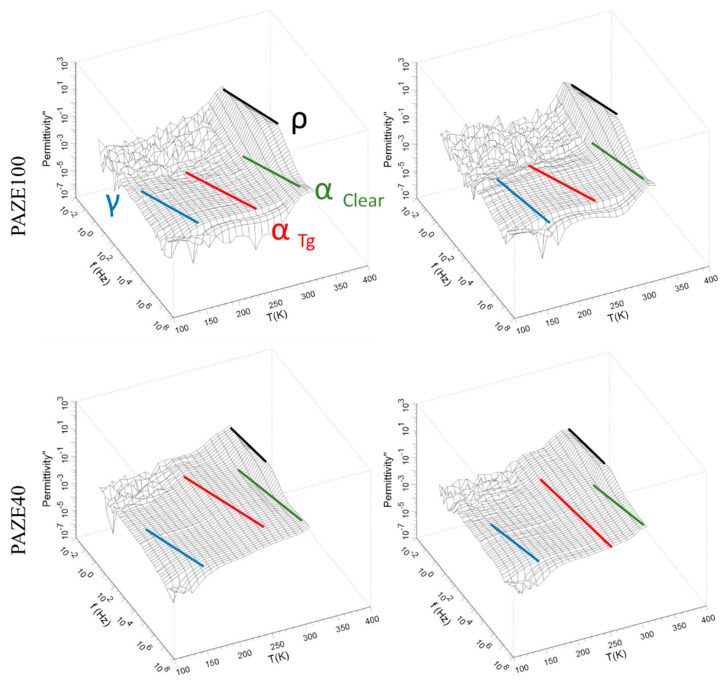
3D plots of imaginary part of dielectric permittivity *ε*″ of the unoriented (**left**) and oriented (**right**) PAZE100 and PAZE40.

**Figure 9:**
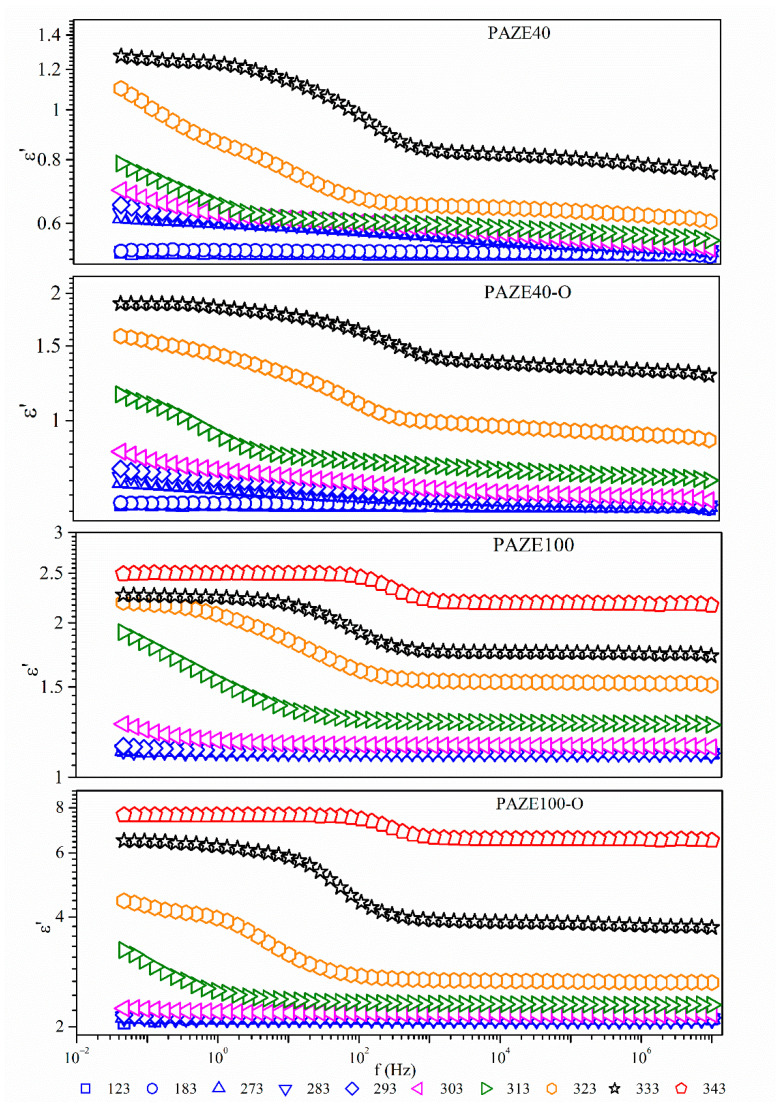
Isothermal curves (in K) of the real part of the dielectric permittivity *ε*′ of the unoriented and oriented PAZE100 and PAZE40.

Figure 8 and Figure 9 show the imaginary part *ε*″ of the complex dielectric permittivity *ε*″ and the isothermal curves of the real *ε*′ and respectively, in a broad range of frequencies of unoriented and oriented membranes. The dielectric relaxation spectrum does not show large differences between the oriented and unoriented membranes as can be seen in Figure 8. In general terms, all these figures exhibit a complex spectrum with broad dielectric relaxation zones. These dielectric relaxations may be related to different molecular motions of the dendritic side groups, or the main chain, as detailed in the following sections and are grouped into two relaxation zones labelled as γ, and *α* in increasing temperature order.

All the analyzed isothermal curves of Figure 9 display a similar pattern, a pronounced plateau, which may be associated with the more prominent relaxation zones labelled as *α.* The plateau corresponding to γ dielectric relaxation is barely discernible. The dielectric permittivity *ε*′ values are higher when the amount of liquid crystalline dendritic units in the structure increases (from 1.4 to 2.5). The orientation of the dendritic lateral chain by the previous heat treatment also increased the values of the *ε*′, especially in PAZE100, (from 2.5 to 8), as decreases the dynamic fragility parameter and increases the free volume near to the glass transition, as seen below.

**Figure 10:**
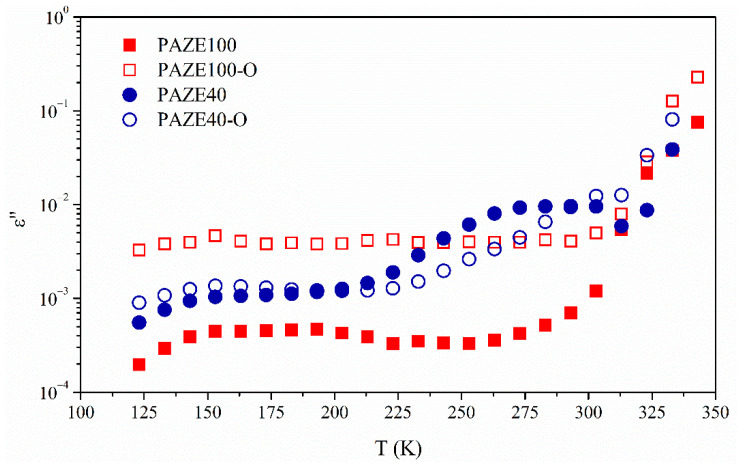
Comparative isothermal curves at 1 kHz of imaginary part of dielectric permittivity of (**b**) unoriented and oriented PAZE100 and PAZE40.

A comparison among the temperature dependence of *ε*″ at the common frequency of 1 kHz is plotted in Figure 10. Dielectric relaxations of the copolymer PAZE40 clearly present the two relaxation zones. However, dielectric relaxation spectra of PAZE 100 are much more complex because different processes occurred overlapped, in agreement with the thermogram of Figure 3, and it is difficult to separate the different relaxation processes. The polyamine main chain of fully modified PAZE100 is supposed to be more rigid than the main chain of PAZE40, due to the higher amount of lateral mesogenic groups. In addition, the presence of a regular structure can enhance the formation of a stable crystalline phase for PAZE100, which consequently affects its polarization.

The imaginary *ε*″ dielectric permittivity versus frequency curves were fitted adding up as many Havriliak–Negami (HN) functions as relaxation processes were necessary to fit all the values for each temperature. A sub-glass transition relaxation labelled as γ-relaxation and a complex dielectric α-relaxation zone were encountered. The α-relaxation zone includes, at least, two close relaxations denominated α_Tg_ and α_Clear_ that can be related to the glass transition and the clearing transition, respectively. The former was not evidenced by DSC and XRD measurements, while the latter could be clearly observed. At the highest temperature and lowest frequencies a *ρ*-Maxwell–Wagner–Sillars polarization phenomenon [36] was observed, which can be related to a conductive effect of displacing dipoles but it has not been analyzed because this work focus only on local molecular mobility from dielectric relaxations.

**Figure 11:**
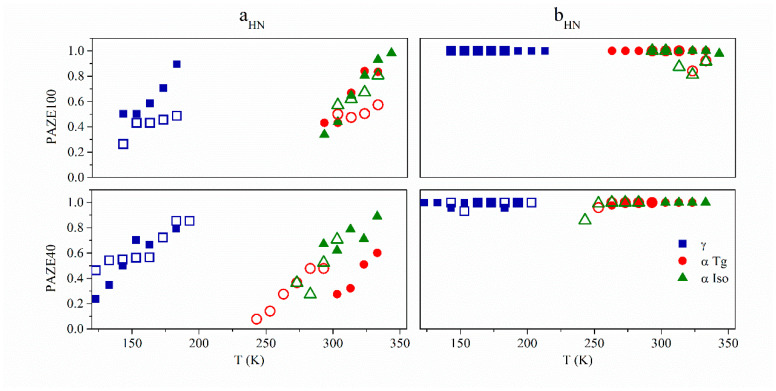
Detail of the Havriliak–Negami parameters *a_HN_* and *b_HN_* for the PAZE100 and PAZE40 for the γ, α_Tg_ and α_Clear_ relaxations of the unoriented (hollow symbols) and oriented (full symbols) copolymers.

The fitting parameters as the dielectric intensity or relaxation strength Δ*ε,* and shape *a_HN_*, *b_HN_* parameters of the Havriliak–Negami equation were calculated for each of the relaxation processes. The relationship between the temperature and each one of the above-mentioned parameters for all materials was analyzed and it is displayed in Table 1 and Figure 11, respectively.

Table 1 shows the dielectric strength below and above the glass transition. As it was expected, the values increased with the temperature. The orientation, due to the crystalline organization of chains, promotes a rather narrow distribution of relaxation times. However, the dielectric strength slightly increased for the α-relaxation zone, especially in the oriented fully modified PAZE100. The melting of the crystalline organization, the glass transition and clearing transition appeared very close. Actually, each process could have a certain influence on the other, and it could shift its position on the temperature axis.

The Havriliak–Negami shape parameters *a_HN_* and *b_HN_*, which were calculated for each system below and above T_g_, are shown in Figure 11. A general review of these parameters indicates that the values of all relaxations increased with increasing temperature. The orientation induced by heat treatment increases these values especially in PAZE100. The *b_HN_* parameters corresponding to all relaxation processes were very close to unity, independent of temperature. This fact indicates the absence of skewness in the high-frequency range of the *ε*″ vs. *ε*′ plot and suggests that the Cole−Cole behaviour at high temperature come close to a semicircle.

### 3.3. Macromolecular Cooperativeness and Thermal Activation of Dielectric Relaxations

**Figure 12:**
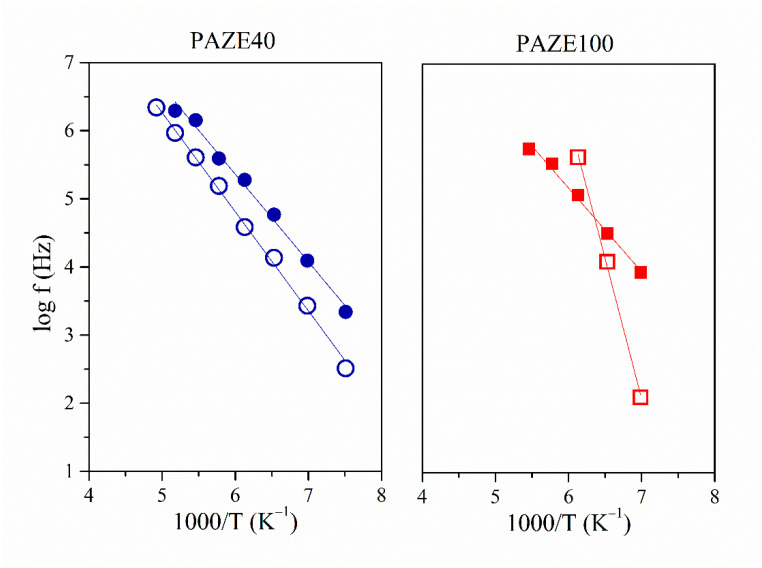
Detail of the Arrhenius maps of the γ relaxation of PAZE100 and PAZE40 for unoriented (full symbol) and oriented (hollow symbol) copolymers.

**Figure 13:**
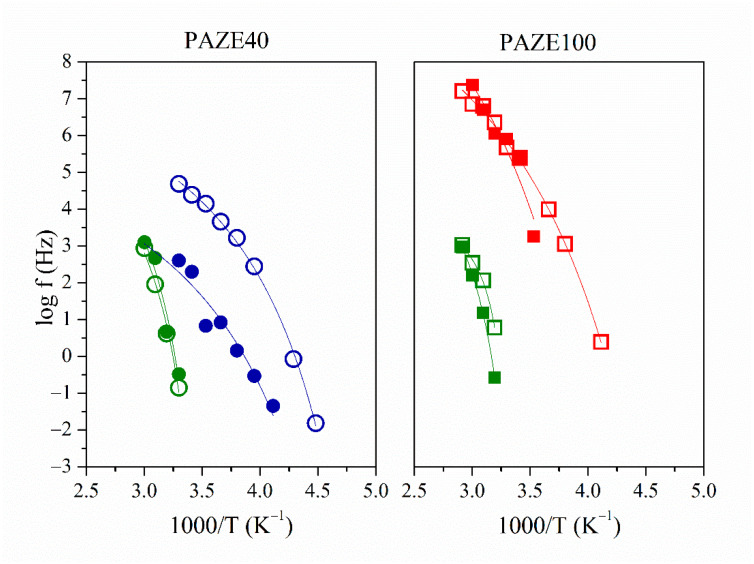
Detail of the Arrhenius maps of the α_Tg_ (circles) and α_Clear_ (triangles) relaxations of PAZE100 and PAZE40 for unoriented (hollow symbols) and oriented (full symbols) copolymers.

The dielectric behaviour of the copolymers was analyzed by means of the Arrhenius plots, which consider the temperature-frequency interdependence for the maxima of loss factor isochrones. The relationship between the relaxation time and the inverse of the temperature (log f vs. T^−1^ plot) shows a linear behaviour. Thus, the dielectric relaxation can be explained by an intramolecular origin, and its thermal activation can be modelled by an Arrhenius-like function. On the other hand, the non-linear dependence between (log f vs. T^−1^) is representative of VFTH-like thermal activations, inherent in intermolecular segmental motions. Figure 12 and Figure 13 show the Arrhenius map for the γ and α relaxation zones, respectively.

In the complex α dielectric relaxation zone, only two close relaxations can be found, according to other reported works [6,7,8,9,10,11,12,13,37,38], at a lower temperature, as the Arrhenius map indicates, the relaxation could be associated with cooperative motions typical of the glass transition and was labelled as α_Tg_ relaxation. It is interesting to mention that the glass transition cannot be observed on the DSC thermograms.

At higher temperatures, the relaxation was ascribed to the clearing transition and labelled as α_Clear_.

**Figure 14:**
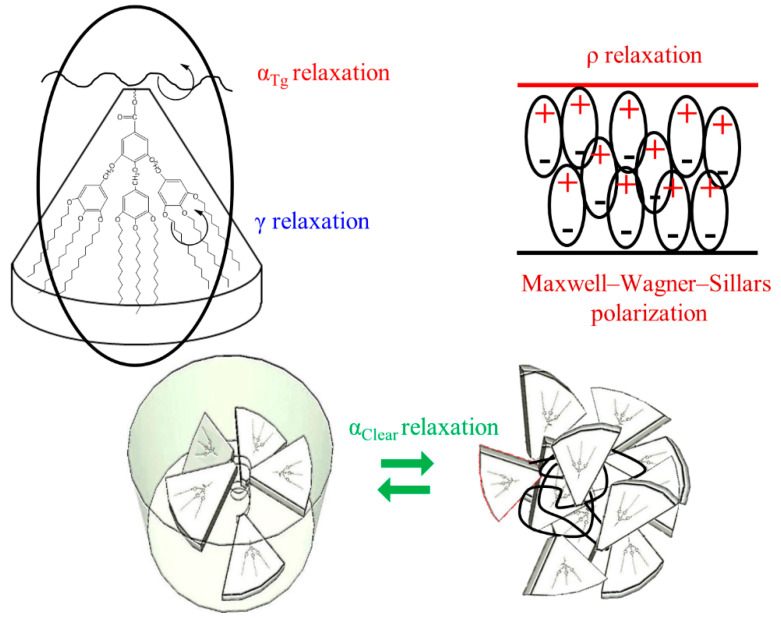
Molecular origin of the different relaxations in PAZE polymer or copolymer.

According to the above results, the molecular origin of the different relaxations is schematized in Figure 14 and the following observations can be raised:

1. The γ relaxation seems to depend only on the benzyloxy terminal group of the mesogenic side groups. It seems to be affected by the orientation of the membrane, especially for the PAZE100: this may be due to the tendency to crystallize this polymer when submitted to annealing.
2. The two relaxations α_Tg_ and α_Clear_, which contribute to the dielectric α relaxation zone, have different behaviour depending on the mesogenic substitution degree. The orientation during preparation did not have an effect on the temperature of α_Clear_ relaxation. The values of α_Clear_ temperature peak at 1 kHz are: 333 K and 330 K for PAZE40 and 337 K for both unoriented and oriented PAZE100 membranes, respectively, probably because the possible crystallization or orientation of the side chains could have already disappeared during the melting, which temperature is lower than the clearing process. On the other hand, the orientation has a perceptible effect on the values of α_Tg_ temperature peak at 1 kHz, especially for the PAZE40, which values increase from 268 K to 320 K for the unoriented and oriented membranes, respectively. This effect is minor in the PAZE 100 which values are 263 K and 270 K, respectively. Unfortunately, this behaviour could not be checked by calorimetric analysis since the glass transition was not visible.

On the other hand, the complexity of the observed relaxations in DETA is also observed by solid-state NMR. Actually, variable temperature ^13^C CP-MAS NMR results indicate, that larger molecular reorientations at the polymer main chain as well as the inner phenyl ring of the dendron are initiated in the same temperature range. This temperature range, where dynamic processes of the polymer main chain are activated, seems to be narrower for PAZE100 as compared to PAZE40, where the motional reduction of the broad polymer main chain signals is a gradual process starting already at a significantly lower temperature.

**Figure 15:**
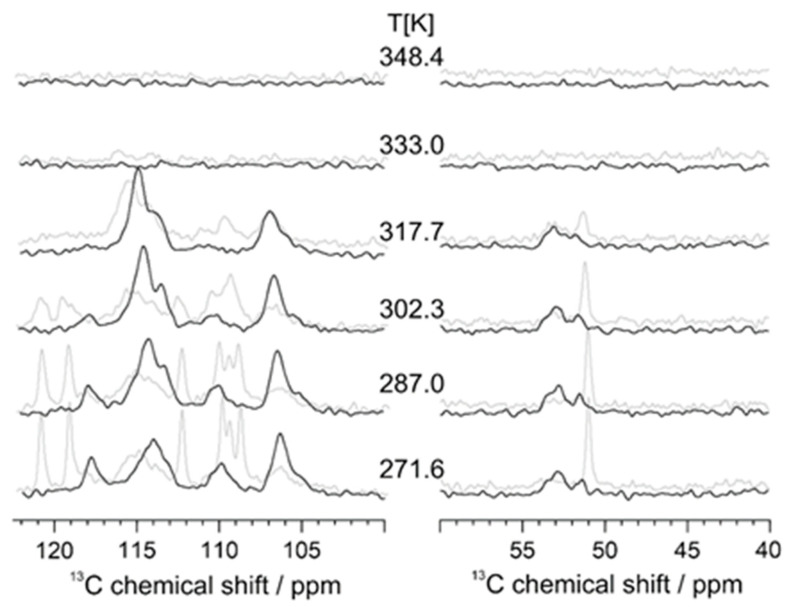
Zoomed ^13^C CP-MAS NMR spectra of thermally treated (black line) and native (grey line) PAZE100 at variable temperature of the outer phenyl rings, inner phenyl ring (**left**) and polymer main chain (**right**).

We tried to elucidate the nature of the molecular motions involved in the relaxations of PAZE100 between 240 and 340 K. Figure 15 shows the temperature dependence of some protonated phenyl sites at the inner and outer phenyl rings as well as the main chain region of [13] ^13^C CP-MAS NMR spectra of PAZE100.

As for the main chain region of the variable temperature 13C CP-MAS NMR spectra (see Figure 15), two NMR signals at 53 ppm and at 51.5 ppm are observed. Upon heating, the 51.5 ppm signal slowly merges with the 53 ppm signal, resulting in a broad featureless NMR signal of the polymer main chain, originating from conformational exchange of the main chain N-CH2 units at a 100 µs time scale. At temperatures above the clearing transition, all CP-MAS signals vanish, because the hetero-nuclear dipolar couplings, needed for the ^1^H-^13^C polarization transfer, are averaged to zero by fast isotropic molecular motions. Considering the behaviour of the dendritic side groups (see Figure 15), the signals of the CH sites (ortho position) of the inner phenyl ring are observed as a single peak at 106 ppm, with a broader component towards lower ppm values and a narrow component at 106.3 ppm, which may arise from better-ordered regions of the sample. With increasing temperature, the signals show a weak tendency to shift towards higher ppm values, suggesting a weakening of the π-stacking of the mesogenic core. Moreover, the narrow signal broadens close to the clearing transition indicating substantial pre-transitional fluctuations of the inner phenyl ring on the sub-millisecond time scale.

The ^13^C NMR signals of the meta-CH sites of the outer phenyl rings of the dendritic side groups indicate that there are phenyl rings in two different packing arrangements seen as peaks at 110 and 118 ppm. In addition to these rigid or slowly moving sites, there are outer phenyl rings (signal at 114 ppm), which rapidly exchange between those two possible packing arrangements with rates above 100 kHz event at the lowest temperature experimentally accessible. With increasing temperature, the signals at 110 and 118 ppm start to broaden and decrease in intensity, while the signal of the exchanging phenyl sites increases.

A closer look to the ^1^H MAS spectrum of the thermally treated PAZE100 recorded at 318 K (see Figure 6c), shows narrow peaks of mobile sites on top of the almost unchanged broad and featureless ^1^H spectrum of the rigid material, indicating the heterogeneous on-set of the transition assigned to α_Tg_ relaxation peak of the DETA analysis. In contrast to the ^13^C CP-MAS spectra, where the signals vanish at the isotropization due to the motional cancellation of hetero nuclear dipolar couplings, ^1^H signals become very sharp and intense with the isotropization transition suggesting a liquid-like behaviour of PAZE100 in this state.

#### 3.3.1. Molecular Origin of the γ Relaxation Zone

The γ relaxation was located at low temperatures 128 K at 1 kHz for both unoriented PAZEs and around 145 K for the oriented samples. The thermal activation was governed by an Arrhenian-like function as shown in Figure 12, with apparent an activation energy Ea around 24 kJ·mol^−1^ for unoriented PAZEs. The orientation process increases the Ea values, especially for PAZE100 that achieves a value of 79 kJ·mol^−1^, as shown in Table 2. Nevertheless, these values are low enough to be characteristic of molecular small-angle reorientations. Therefore, this relaxation process was ascribed to intramolecular mobility of the local secondary phenyl-aliphatic chain (R- in Figure 1) of the dendritic liquid crystal. The orientation process provides a more ordered arrangement of polymer structure, where local π-stacking arrangements between neighboring outer phenyl rings are optimized so that the apparent activation energy to induce this motion is substantially increased, especially in fully modified PAZE100 with the highest density of dendritic side groups.

#### 3.3.2. Macromolecular Origin of the α Relaxation Zone

At higher temperatures, a high intensity and complex dielectric relaxation zone is observed, which could be related to three almost overlapping processes:

1. Molecular precursor motions of the melting of a certain crystalline portion of the lateral dendritic chains;
2. The associated motion to the glass transition;
3. Molecular precursor motions of the clearing transition. However, only two dielectric relaxations can be clearly distinguished: one with lower intensity ascribed to the glass transition, i.e., α_Tg_, because the percentage of crystallinity is indeed very small, as shown by DSC and XRD results, and should be overlapped by the glass transition processes; the other one much more prominent related to clearing transition, i.e., α_Clear_.

The α_Tg_ dielectric relaxation was explained in terms of VFTH-like function as it was can be ascribed to intermolecular motions related to the glass transition, as Figure 13 shows for unoriented and oriented systems.

Table 3 shows the values of the dynamic fragility parameter D, which physical interpretation is a measure of deviation from the Arrhenius temperature dependence of relaxation times. On the other hand, D also could quantify the steepness of the dielectric jump of the dielectric permittivity near the glass transition [39] and it depends on the cooperativity of the dynamic processes [39,40,41]. Although, from the Angell classification, the obtained D values are low enough to correspond to a fragile system, the dynamic fragility parameter D has a higher value for PAZE100 than for PAZE40.

The differences found between both polymers as a consequence of the substitution of the side chain (PAZE100 or PAZE40) or those due to orientation, are not evident or easy to interpret since, as indicated by the NMR results, different molecular reorganizations in the range of temperatures between 290 and 333 when the clearing transition occurs is very complex.

The values of D seem to indicate that the PAZE40 copolymer maintains better the locally ordered structure near the glass transition than the PAZE100 polymer; this, in turn, may be due to the number of polymer repetitive units included in the helical structure that is 10.5 in PAZE40, versus the 4.6 ones that take part to the helices in the case of PAZE100. Thus, the structures of PAZE100 could change rapidly when the temperature approaches the glass transition and quickly leave the ordered glass structure. Nevertheless, the orientation produces a different effect on PAZE100 and PAZE40. For one thing, the temperature of the α_Tg_ dielectric relaxation peak at 1 kHz increases from 268 K to 320 K for PAZE 40 while the dynamic fragility parameter D almost stays constant. Unfortunately, these changes cannot be verified in the calorimetric thermogram since the glass transition is not perceptible. For another thing, the temperature of the α_Tg_ dielectric relaxation peak at 1 kHz for the oriented PAZE100 hardly changes (from 263 to 270 K), whereas the value of D decreases, even below the values obtained for the PAZE40. The results that the NMR of thermally treated PAZE100 can provide indicate that two distinct back-conformations start to exchange at temperatures just below the clearing transition. At the same temperature, the ^1^H MAS NMR spectrum of the thermally treated PAZE100 shows a heterogeneous behavior below the clearing transition, meaning some side chains are already quite mobile, whereas others are still very rigid. This heterogeneity of the side chain dynamics is needed for the conformational exchange, as in the fully substituted PAZE100 the main chain cannot change conformations without side chain reorganization. These results may be in line with the values obtained for the free-volume coefficients *Φ_B_* and the thermal expansion coefficients α_f_ near the glass transition for the oriented PAZE100 that are much higher. However, these values slightly change with the orientation in PAZE40. Thus, the previous thermal treatment would provide a possibility to change the mobility of these materials, especially when approaching the glass transition.

The α_Clear_ relaxation is related to a precursor motion of a supramolecular phenomenon, typical of thermotropic liquid crystals, based on the predominance of thermal motions over the weak interactions between the dipole-type molecules or dispersion forces that determine the arrangement into a liquid crystalline phase. These interactions are strong enough to maintain associations between molecules in a preferred orientation, though they keep their freedom to move, since they are not covalently bound. This supramolecular phenomenon corresponds to the disassembling of the polymeric columns and provokes the disappearance of the liquid crystalline order.

The α_Clear_ relaxation was also explained by a VFTH-like function, which means that it is an intermolecular motion. Table 4 gathers the results of fitting the thermal activation of the relaxation times for oriented and unoriented copolymers. As it is expected, both oriented and unoriented PAZE40 have a TVFTH temperature related to the clearing relaxation lower than PAZE100. In both cases, the grafted tapered moieties form layers with their neighbors that are arranged at different angles, giving a liquid crystal with a helical arrangement. Thus, these moieties are placed parallel, but at the same time, they can move one each other along their axes. When the temperature is raised above the clearing point, thermal motions prevail over dispersion forces, and the polymeric material undergoes a first-order transition and turns isotropic. For the α_Clearing_ relaxation, the parameter D exhibits a very low value and the orientation does not have any effect on both materials. In general, it is possible to suppose that orientation during the preparation of the membranes did not have any effect on this relaxation process.

## 4. Conclusions

DSC, XRD and NMR studies show that all the studied native unoriented membranes based on dendronized polymers and copolymers obtained by chemical modification of poly[2-(aziridin-1-yl) ethanol] (PAZE) with the dendron 3,4,5-tris[4-(n-dodecan-1-yloxy)benzyloxy]benzoate show a liquid crystal mesophase. When the available free volume is sufficient, these polymers partially organize into crystalline phases. In the case of fully modified PAZE100, high regularity of the structure could favor organization of the polymer into crystalline domains; nevertheless, the rigidity of the structure and the dense packing of the dendritic side-chains prevents the occurring of crystalline structures directly on cooling from the molten state. The thermal treatment enhances nano-crystallinity with significant dynamic heterogeneities. In the case of samples with partial modification degrees such as PAZE40, annealing seems to favor the organization into a more stable LC phase, while crystalline portions tend to disappear.

The dielectric broadband spectroscopy spectrum of the dendronized PAZE100 and PAZE40 membranes are composed of two dielectric relaxation zones: (i) an intramolecular γ relaxation that was associated with the orientation of the benzyloxy substituent of the dendritic group; (ii) a complex intermolecular and cooperative α relaxation zone, related to, at least two distinct relaxation processes, the glass (α_Tg_) transition (main chain relaxation) and the clearing (α_Clear_) processes.

The complexity of the observed relaxation processes in DETA could be also guessed by solid-state NMR, which results indicate that larger molecular reorientations at the polymer backbone as well as the inner phenyl ring of the dendron are initiated within a very close temperature range. The dynamic processes of the polymer backbone are activated for PAZE100 in a narrower temperature window close to the clearing transition as compared to PAZE40, where the motional reduction of the broad polymer backbone signals is a gradual process starting already at a lower temperature. When the temperature increases, the narrow signals of the inner phenyl ring broaden close to the clearing transition indicating pre-transitional fluctuations of the inner phenyl ring on the sub-millisecond time scale. The NMR signals of the meta-CH sites of the outer phenyl rings of the dendritic side groups indicate that there are phenyl rings in two different packing arrangements (signals at 110 and 118 ppm) as well as phenyl rings (signal at 114 ppm), which exchange between those two possible packing arrangements.

The orientation procedure during the preparation of the membranes does not affect the clearing process. However, it increases the apparent activation energy of the γ relaxation and, consequently, changes the molecular mobility of the dendritic side groups, especially in PAZE100. In the less substituted copolymer, PAZE40, this behavior is significantly weaker. This aspect would be relevant in terms of the stability of the nano-channels, which are necessary for ion transport.

Grafting with dendritic groups to the backbone structure increases the dynamic fragility parameter near the glass transition of PAZE100. Therefore, PAZE100 can change the organization of the polymer backbone in a narrow temperature window close to the glass transition temperature. The orientation procedure for the membranes did not change the fragility of PAZE40 but shifted the temperature of the α_Tg_ relaxation peak towards higher temperatures, confirming the stabilization of the dendritic structure.

The addition of dendritic groups and the previous orientation by thermal treatment offers the possibility of designing PAZE-based polymers with the desired fragility and sufficient stabilization of the dendritic chains, which can turn these polymers into efficient membranes for ionic transport applications.

## Figures and Tables

**Table 1:** Detail of Δε obtained from the fitting of the Havriliak–Negami equation.

**Δ ε**	**PAZE40**				**Δ ε**	**PAZE100**			
	T (K)	153	163	173		T (K)	153	163	173
	γ	0.001	0.001	0.002		γ	0.001	0.001	0.001
	γ-O	0.005	0.003	0.002		γ-O	0.016	0.019	0.018
	T (K)	253	263	273		T (K)	293	303	313
	α_Tg_	0.049	0.051	0.087		α_Tg_	0.003	0.004	0.006
	α_Tg_-O	0.017	0.022	0.041		α_Tg_-O	0.007	0.007	0.020
	T (K)	293	303	313		T (K)	313	323	333
	α_Clear_	0.080	0.176	0.211		α_Clear_	0.242	0.252	0.312
	α_Clear_-O	0.083	0.083	0.098		α_Clear_-O	1.197	1.409	1.926

**Table 2:** Apparent activation energies and Tmax of the γ relaxations of oriented and unoriented PAZE100 and PAZE40, as obtained by the fitting of the Arrhenius equation. Margins within 3% of standard deviation.

Material	logf_0_ (Hz)	Ea (kJ·mol^−1^)	T_max_ 1 kHz (K)	R^2^
PAZE100	12.5	23.5	128.6	0.99
PAZE100-O	30.9	78.9	147.5	0.99
PAZE40	13.1	24.6	127.6	0.99
PAZE40-O	15.4	33.9	143.5	0.97

**Table 3:** VFTH parameters of α_Tg_ relaxation for un-oriented and oriented PAZE100 and PAZE40.

α_Tg_	τ_0_ (s)	D	T_VFTH_ (K)	Ф_Tg_	α_Tg_ × 10^4^ (K^−1^)	R^2^
PAZE100	11.8 ± 0.4	8.5±1.4	180 ± 6	0.033	6.5	0.99
PAZE100-O	8.6 ± 0.7	3.9 ± 0.5	180 ± 6	0.072	14.3	0.94
PAZE40	8.1 ± 0.4	5.0 ± 0.8	183 ± 4	0.055	11.0	0.99
PAZE40-O	6.1 ± 0.5	5.7 ± 0.5	184 ± 4	0.048	9.5	0.94

**Table 4:** VFTH parameters of α_Clear_ relaxation for unoriented and oriented PAZE100 and PAZE40.

α_Clear_	τ_0_ (s)	D	T_VFTH_ (K)	Ф_Tg_	α_Tg_ × 10^4^ (K^−1^)	R^2^
PAZE100	4.9 ± 0.8	1.4 ± 0.2	278	---	---	0.92
PAZE100-O	7.1 ± 0.2	2.1 ± 0.1	278	0.086	17.2	0.99
PAZE40	7.2 ± 0.5	2.5 ± 0.2	268	0.075	15.1	0.98
PAZE40-O	7.6 ± 1.0	2.5 ± 0.4	268	0.075	15.0	0.93

## Data Availability

The data presented in this study are available on request from the corresponding author. The data are not publicly available due to their deposition at an offline disk, MEMTEC group, URV, Tarragona, Spain, and UPV, Valencia, Spain.
